# Association between ankle–brachial index and body composition parameters measured by bioelectrical impedance analysis in patients with high-risk type 2 diabetes mellitus: a cross-sectional study

**DOI:** 10.1186/s12902-026-02488-4

**Published:** 2026-08-14

**Authors:** Faezeh Mansouri-Roudi, Solmaz Hasani, Shohre Vojoohi, Masoumeh Sadeghi, Mostafa Afsharianfar

**Affiliations:** 1 Department of Internal Medicine, Imam Reza Hospital, Mashhad University of Medical Sciences, Mashhad, Iran; 2 Metabolic Syndrome Research Center, School of Medicine, Mashhad University of Medical Sciences, Mashhad, Iran; 3 Department of Internal Medicine, Aria Hospital, Mashhad, Iran; 4 Department of Epidemiology, Faculty of Health, Mashhad University of Medical Sciences, Mashhad, Iran; 5 Simorgh Diabetes Clinic, Mashhad, Iran

**Keywords:** Body composition, Bioelectrical impedance analysis, Ankle-brachial index, Peripheral arterial disease, Type 2 diabetes mellitus

## Abstract

**Background:**

Peripheral arterial disease (PAD) is a common macrovascular complication of type 2 diabetes mellitus (T2DM) associated with substantial morbidity. The ankle-brachial index (ABI) is a simple, non-invasive, and widely recommended tool for PAD screening, while alterations in body composition may influence cardiovascular risk. This study investigated the association between bioelectrical impedance analysis (BIA)-derived body composition parameters and ABI in patients with T2DM.

**Methods:**

In this cross-sectional study, 100 high-risk adults with type 2 diabetes mellitus attending a tertiary diabetes clinic were consecutively recruited between December 2024 and December 2025. Peripheral arterial disease (PAD) was assessed using the ankle-brachial index (ABI), and body composition was measured by multi-frequency bioelectrical impedance analysis (BIA). Demographic, clinical, and laboratory data were also collected. Comparisons of body composition parameters across ABI categories were performed using non-parametric tests. Multivariable binary logistic regression analyses were conducted to evaluate the independent associations of representative body composition parameters with PAD after assessment of multicollinearity. Adjusted odds ratios (ORs) with 95% confidence intervals (CIs) were calculated, with p-Value < 0.05 considered statistically significant. All analyses were conducted using R statistical software for Windows (v 4.5.2, R Core Team 2025).

**Results:**

Body composition parameters did not differ significantly across ABI categories (p-Value > 0.05). Patients with PAD had lower HDL-C and higher TSH levels than those without PAD (p-Value = 0.02 and p-Value = 0.01, respectively). In separate multivariable logistic regression models adjusted for age and hypertension, neither muscle mass nor visceral fat area showed an independent association with PAD.

**Conclusion:**

Representative BIA-derived body composition parameters showed no independent association with PAD in high-risk patients with T2DM. Given the limited number of PAD events, these findings should be interpreted cautiously and confirmed in larger, adequately powered prospective studies.

**Clinical trial number:**

Not applicable.

## Introduction

Peripheral artery disease (PAD) is a progressive atherosclerotic disorder affecting the arteries of the lower extremities and represents a major global health burden, particularly among patients with type 2 diabetes mellitus (T2DM) [1, 2]. The prevalence of PAD increases markedly with age and is strongly associated with traditional cardiovascular risk factors such as smoking, hypertension, and diabetes [2, 3]. Among individuals with T2DM, approximately 14% to 20% are affected, and the disease is associated with substantially higher risks of limb amputation, prolonged hospitalization, and mortality [4–7].

Despite its clinical significance, PAD remains substantially underdiagnosed because more than half of affected patients, and up to 80% of those with T2DM, are asymptomatic [8]. Moreover, conventional cardiovascular risk prediction models have demonstrated limited accuracy in identifying individuals at risk for PAD [9–12]. Therefore, current international guidelines recommend proactive screening strategies that incorporate clinical risk assessment together with the ankle-brachial index (ABI), a simple, non-invasive, and reliable tool for the diagnosis of PAD (13, 14). The ankle-brachial index (ABI) is calculated as the ratio of the higher systolic blood pressure measured at the ankle (dorsalis pedis or posterior tibial artery) to the higher systolic brachial pressure [15]. The reference standard for measuring the ankle-brachial index (ABI) is Doppler ultrasonography, which involves sequential measurement of systolic blood pressure in all four limbs [15]. Despite its diagnostic value, this technique is relatively time-consuming, requires trained personnel [16, 17]. However, the 2012 American Heart Association statement emphasized the need for simpler and more efficient methods for ABI assessment [15]. Consequently, automated oscillometric devices have emerged as an attractive alternative, offering rapid, fully automated measurements that minimize observer-related variability and eliminate the need for specialized operator training [18, 19].

Growing evidence indicates that body composition, particularly adipose tissue distribution, plays an important role in the development of atherosclerosis. Excess visceral adiposity has been associated with endothelial dysfunction, insulin resistance, systemic inflammation, and carotid atherosclerosis, whereas greater subcutaneous fat accumulation may exert protective vascular effects in patients with T2DM [20–24]. Furthermore, adipose tissue may contribute to thrombotic processes by promoting coagulation cascade activation and platelet activity, thereby facilitating the development and progression of atherosclerotic disease [25, 26].

Bioelectrical impedance analysis (BIA) is a widely used, non-invasive technique for estimating body composition by measuring the body’s electrical impedance in response to a painless, low-intensity alternating electrical current [27]. Because electrical conductivity differs among lean tissue, adipose tissue, and body water compartments, BIA enables the estimation of body composition parameters such as fat mass, fat-free mass, skeletal muscle mass, total body water, and phase angle. Owing to its rapid acquisition, ease of use, portability, and relatively low cost, BIA has become a practical alternative to more sophisticated imaging modalities, including dual-energy X-ray absorptiometry (DXA), computed tomography (CT), and magnetic resonance imaging (MRI) [28]. The technique operates on the principle of measuring the body’s impedance -a composite of resistance (R) and reactance (Xc), expressed as Z² = R² + Xc²- to a low-level electrical current, which allows for the estimation of total body water and, subsequently, a two-compartment model distinguishing between fat mass and fat-free mass using validated prediction equations [29].

Several BIA technologies have been developed to improve measurement accuracy and broaden clinical applications. Conventional single-frequency BIA (SF-BIA) operates at a fixed frequency, typically 50 kHz, and provides estimates of fat mass, fat-free mass, skeletal muscle mass, and total body water using validated predictive equations [27]. In contrast, multifrequency BIA (MF-BIA) utilizes multiple electrical frequencies to distinguish intracellular from extracellular water, thereby providing a more comprehensive assessment of body composition and hydration status [27, 30]. More advanced techniques include bioelectrical impedance spectroscopy (BIS), which measures impedance across a wide spectrum of frequencies, and bioelectrical impedance vector analysis (BIVA), which interprets raw impedance measurements without relying on predictive equations and provides qualitative information on hydration and cellular integrity [27, 31–33].

BIA devices also differ in electrode configuration and measurement methodology. Consumer-grade devices commonly employ bipolar hand-to-hand or foot-to-foot configurations, whereas clinical-grade systems generally use tetrapolar or octopolar electrode arrangements to improve measurement precision and reproducibility [34–37]. Octopolar devices additionally allow segmental analysis of the trunk and individual limbs, facilitating more detailed assessment of regional body composition [38, 39]. Regardless of the technology used, accurate BIA measurements require standardized testing conditions, including appropriate hydration status, fasting, consistent body positioning, and the use of validated predictive equations to ensure reliable and reproducible results [27].

According to the American Diabetes Association’s 2026 Standards of Care as well as other highly endorsed guidelines, the ABI remains the recommended non-invasive screening tool for peripheral arterial disease, while body composition parameters such as visceral adiposity are also recognized contributors to cardiovascular risk [8, 40, 41]. Because both ABI and BIA-derived indices independently reflect cardiovascular risk, investigating their association may improve vascular risk stratification in patients with type 2 diabetes. A growing body of literature has been emerging with promising findings [42, 43].

Accordingly, vascular complications are one of the most concerning subjects among patients with T2DM, generally deteriorating along with disease progression and the presence of metabolic syndrome. Despite the clinical importance of the body composition parameters and the ABI, research on their relation is remarkably limited. This leaves a considerable gap in understanding whether detailed body composition profiles could inform cardiovascular risk or elucidate links between body composition and vascular health. Therefore, this study aimed to investigate the possible relation between the body composition parameters and the ABI in patients with T2DM.

## Materials and methods

### Study design and setting

In this quantitative cross-sectional study, participants were recruited from patients attending the Internal Medicine Clinic at Imam Reza Hospital in Mashhad from December 2024 to December 2025.

## Study participants and sampling

Adult patients (≥ 18 years) with type 2 diabetes mellitus (T2DM) were invited to participate. Participants were recruited consecutively via convenience sampling over a 12-month period to reach the intended sample size.

## Inclusion and exclusion criteria

High-risk diabetic patients were defined by the following criteria and included in this study. Eligible patients were referred to the Simorgh Diabetes Clinic for subsequent assessments. Patients with any of the following criteria were included in our study: age ≥ 50 years; documented T2DM duration > 10 years; presence of microvascular complications (neuropathy, retinopathy, or nephropathy); clinical evidence of diabetic foot complications (i.e. foot ulcers); or diabetes-related organ dysfunction.

## Sample size calculation

A sample size calculation was performed using G*Power version 3.1.9 software [44, 45]. For a multivariable logistic regression model aiming to detect a moderate effect size (f² = 0.2) with 10 predictor variables, a type I error (α) of 0.05, and a power (1-β) of 0.80, a minimum sample of 91 participants was required. Accounting for an estimated 10% dropout in the patient referral process, a final target sample of 100 participants was set.

## Data collection and measurements

The primary outcome was the Ankle-Brachial Index (ABI), measured separately for the right (ABI-R) and left (ABI-L) limbs. The ABI was measured for all participants using the AngE™ ABI+ oscillometric device (SOT medical systems, Maria Rain, Austria). Measurements were performed in a supine position following a standard protocol, with systolic blood pressures recorded from both brachial arteries and from the dorsalis pedis and posterior tibial arteries of each ankle. The ABI for each limb was calculated as the ratio of the higher ankle pressure to the higher brachial pressure. The body composition was evaluated through segmental assessment method using multiple frequencies (10 kHz, 50 kHz, 100 kHz, and 250 kHz) at morning and while fasting, using a device with a tetra-polar 8-point tactile electrode system (Mediana i35, Mediana Co., Seoul, South Korea). The measured parameters included fat-free mass (FFM), muscle mass (MM), total body water (TBW), waist circumference (WC), and specific adipose tissue indices including abdominal fat ratio (AFR), visceral fat area (VFA), subcutaneous fat area (SFA), and their ratio (VSR). Data on the demographics (age, sex, height, weight) and diabetes-specific variables (disease duration, fasting blood sugar [FBS], glycated hemoglobin [HbA1c]) were collected. A comprehensive panel of laboratory assays was performed to characterize the cardiometabolic and renal risk profile. These assays included lipid parameters (total cholesterol, HDL, LDL, triglycerides), renal function markers (BUN, creatinine, e-GFR, albuminuria), liver enzymes (AST, ALT, ALP), a complete blood count (WBC, Hb, Platelets count), thyroid-stimulating hormone (TSH), and vitamin D levels. Eventually, relevant medical history, including past medical history, family history of diabetes/cardiovascular disease, and prior surgical interventions, was also documented.

### Statistical analysis

Categorical variables are presented as frequencies and percentages. The parametric variables were reported as mean ± standard deviation (SD) along with the minimum-maximum ranges. The non-parametric variables were demonstrated as median with Q1-Q3 range. Kolmogorov-Smirnov and Shapiro-Wilk tests were used to determine the normal distribution of the continuous variables. The difference of frequencies across the ABI subgroups were analyzed using the Chi-square test. Findings were demonstrated based on the ABI subgroups. According to current guidelines, ABI of 0.91–0.99 is generally considered borderline while 1.00–1.30 is considered normal. However, for analytical purposes and because of the limited sample size, participants with borderline (0.91–0.99) and normal (1.00–1.30) ABI values were analyzed as a single reference group. Therefore, the association between the body composition parameters and ABI was assessed in two separate conditions: primarily, the mean differences of body composition parameters were evaluated across three ABI subgroups using Kruskal-Wallis test. On the next step, after omitting the patients with ABI > 1.3, the mean differences of body composition parameters were again analyzed using Mann-Whitney U test across two remaining subgroups.

To identify body composition parameters independently associated with peripheral arterial disease (PAD), multivariable binary logistic regression analyses were performed using PAD (presence/absence) as the dependent variable. Prior to model construction, multicollinearity among body composition variables was assessed using variance inflation factors (VIFs). Because several body composition parameters demonstrated substantial collinearity, they were not entered simultaneously into the same regression model. Instead, representative variables from different physiological domains were selected based on previous studies, and separate parsimonious multivariable logistic regression models were constructed [46, 47]. Model A, the lean body mass representing model, included age, hypertension, and muscle mass (MM), whereas Model B, the adiposity representing model, included age, hypertension, and visceral fat area (VFA). The Enter method was used for all analyses. Adjusted odds ratios (ORs) with 95% confidence intervals (95%CIs) were calculated, and a two-sided P value < 0.05 was considered statistically significant. Data were managed and analyzed using R statistical software for Windows (v 4.5.2, R Core Team 2025).

## Results

### General characteristics

The study population comprised 100 individuals diagnosed with T2DM. The mean age was 62.89 ± 8.53. Forty-eight (48%) were male and 52 (52%) were female. The mean duration of diabetes was 13.41 ± 8.19 years, ranging from 1 to 45. The mean right and left ABI values were 1.10 ± 0.19 and 1.06 ± 0.20, respectively. No patient was included due to diabetic foot complications (i.e. foot ulcer).

### Main results

The results indicate that the mean ABI for 77% of the study population fell outs the normal range, leaving only 11% of patients with probable PAD (Table 1).

**Table 1:** Demographic and clinical characteristics of the study population

Variables	Total (*n* = 100)	ABI			*p*-Value
		< 0.9 (*n* = 11)	0.91–1.3 (*n* = 77)	> 1.3 (*n* = 12)	
Age^a^ (years)	62.89 ± 8.53 (44–96)	62.82 ± 11.64 (49–80)	62.65 ± 8.41 (44–96)	64.50 ± 6.30 (54–72)	0.78*
Male^b^	48 (48.00)	6 (54.50)	36 (46.80)	6 (50.00)	0.88^¶^
ABI-R^c^	1.08 (0.99–1.19)	0.84 (0.81–0.91)	1.08 (1.01–1.17)	1.46 (1.26–1.54)	< 0.001^§^
ABI-L^c^	1.02 (0.96–1.14)	0.83 (0.75–0.85)	1.02 (0.98–1.11)	1.40 (1.19–1.71)	< 0.001^§^
Weight^a^ (kg)	74.38 ± 12.69 (51.6-110.4)	70.24 ± 9.91 (54.6-110.4)	74.33 ± 13.01 (47.5–111.0)	78.46 ± 12.47 (63.3–93.0)	0.30*
Height^a^ (cm)	162.82 ± 10.04 (138–187)	161.09 ± 10.79 (142.0-178.0)	162.88 ± 10.29 (138.0-187.0)	164.04 ± 8.19 (152.0-185.0)	0.78*
BMI^a^ (kg/m^2^)	28.14 ± 4.49 (18.28–46.20)	27.43 ± 4.26 (21.06–38.20)	28.09 ± 4.45 (18.28–46.20)	29.28 ± 5.06 (20.28–38.71)	0.55*
Waist Circumference^c^ (cm)	90.5 (86.2–97.0)	88.7 (86.93-96)	91.1 (85.9–97)	90.5 (86.3-100.8)	0.29^§^
SBP^c^ (mmHg)	120 (110–130)	117.5 (115–135)	120 (110-131.5)	112.5 (10.8.5-121.5)	0.72^§^
DBP^c^ (mmHg)	80 (70–85)	77.5 (75-88.5)	80 (70–85)	75 (68.5–80)	0.14^§^
Diabetes duration^c^ (years)	12 (8–20)	13 (8–25)	12 (8–18)	11.50 (5.75–20.75)	0.48^§^
Smoking^c^	5 (5.00)	0 (0.00)	3 (3.90)	2 (16.70)	0.19^¶^
HTN^c^	52 (52.00)	7 (63.60)	35 (45.50)	10 (83.30)	0.03^¶^
Thyroid^c^	20 (20.00)	3 (27.30)	15 (19.50)	2 (16.70)	0.83^¶^
Dyslipidemia^c^	6 (6.00)	0 (0.00)	5 (6.50)	1 (8.30)	0.99^¶^
Cancer^c^	5 (5.00)	0 (0.00)	5 (6.50)	0 (0.00)	0.99^¶^
Cardiac events^c^	19 (19.00)				
Angioplasty	13 (13.00)	1 (9.10)	9 (11.70)	3 (25.00)	0.67^¶^
Angioplasty & MI	6 (6.00)	1 (9.10)	5 (6.50)	0 (0.00)	
Surgical History^c^	19 (19.00)	1 (9.10)	14 (18.20)	4 (33.30)	0.36^¶^
Family history of disease^c^	77 (77.00)				
Diabetes	46 (46.00)	4 (36.40)	40 (51.90)	2 (16.70)	0.05^¶^
HTN	9 (9.00)	0 (0.00)	7 (9.10)	2 (16.70)	
Diabetes & HTN	20 (20.00)	1 (9.10)	14 (18.20)	5 (41.70)	
Cancer^**c**^	2 (2.00)	1 (9.10)	1 (1.30)	0 (0.00)	

^a^ mean ± standard deviation (minimum-maximum) ^b^ n (%) ^c^ median (Q1-Q3) * One-way ANOVA ^¶^ Chi-square tests ^§^ Kruskal-Wallis testABI-R, Ankle Brachial Index- right arm and right leg; ABI-L, Ankle Brachial Index- left arm and left leg; BMI, Body Mass Index; SBP, Systolic Blood Pressure; DBP, Diastolic Blood Pressure; HTN, Hypertension

A history of hypertension was reported by 52 patients (52%), while 20% reported a history of thyroid disease, 19% reported a history of cardiovascular events, and 19% reported a history of surgery. The demographic characteristics of the study population are presented in Table 1. Only one patient (1%) had undergone peripheral vascular surgery, and 7% reported a history of coronary artery bypass surgery. Other surgical histories included thyroid lobectomy (2%), hysterectomy (8%), and mastectomy (3%). The distribution of demographic and clinical variables was similar across ABI groups, with no statistically significant differences except for a history of hypertension.

Analysis of laboratory parameters (Table 2) showed comparable and statistically similar levels of FBS and HbA1c across all three ABI subgroups. Renal function and liver enzyme levels followed a similar pattern. Although higher TSH levels were noted in patients with abnormal ABI (either < 0.9 or > 1.3), the difference was not statistically significant. The lipid profile analysis suggested a trend toward an atherogenic pattern in the low ABI subgroup (< 0.9), characterized by higher triglyceride and lower HDL-C levels. However, no statistically significant differences in triglyceride or LDL-C levels were found between the ABI subgroups.

**Table 2:** Biochemical features of the study population (For all individuals and by ankle-brachial index category)

Lab Features	Total (*n* = 100)	ABI (Ankle-Brachial Index), Mean ± SD			*p*-Value
		< 0.9 (*n* = 11)	0.91–1.3(*n* = 77)	> 1.3 (*n* = 12)	
FBS^a^ (mg/dL)	127.00 (109.00-154.00)	136.50 (92.75-151.75)	126.50 (109.75-156.25)	116 (101.50–147)	0.78*
HbA1C^a^ (%)	6.40 (6.40–7.87)	7.10 (6.52–7.72)	6.90 (6.30–7.90)	6.80 (6.37–8.35)	0.98^¶^
Hemoglobin^a^ (g/dL)	14.00 (12.90–15.20)	15.10 (12.62–15.92)	14.00 (12.92–15.07)	13.00 (12.00-14.80)	0.16^¶^
BUN^a^ (mg/dL)	17.50 (14.00–21.00)	17.00 (13.95–25.75)	17.50 (14.00–21.00)	18.00 (13.12–23.75)	0.66^¶^
Creatinine^a^ (mg/dL)	1.00 (1.00-1.20)	1.08 (0.90–1.24)	1.00 (0.81–1.20)	0.95 (0.69–1.10)	0.75^¶^
Total-C^a^ (mg/dL)	138.00 (125.00-160.00)	130.50 (118.50-159.75)	140.00 (127.75-161.25)	132.00 (105.50–153)	0.93^¶^
HDL-C^a^ (mg/dL)	44.50 (36.75-49.00)	36.50 (31.25–41.50)	46.00 (38.00–52.00)	45.00 (28.60–51.00)	0.03^¶^
LDL-C^b^ (mg/dL)	75.26 ± 22.16 (28.00-127.00)	75.26 ± 22.16 (37.00-122.00)	75.89 ± 20.44 (28.00-127.00)	70.05 ± 29.09 (38.00-107.00)	0.80*
Triglycerides^a^ (mg/dL)	124.00 (96.00-155.00)	134.50 (107.50-312.25)	123.00 (97.00-153.00)	113.00 (69.00-200.00)	0.64^¶^
25-OH Vit D^a^ (ng/mL)	35.60 (28.00–45.00)	29.40 (26.00-33.75)	37.85 (28.00–48.00)	30.32 (19.27–36.25)	0.61^¶^
AST^a^ (mg/dL)	20.00 (16.00–24.00)	21.50 (14.50–28.80)	20.00 (17.00–24.00)	20.00 (14.50–23.00)	0.16^¶^
ALT^a^ (mg/dL)	20.00 (15.00–29.00)	28.50 (20.25-37.00)	20.00 (15.00–27.00)	18.00 (14.50–34.00)	0.58^¶^
ALP^b^ (mg/dL)	169.73 ± 66.21 (54.00-346.00)	191.56 ± 57.64 (54.00-273.00)	165.55 ± 68.71 (54.00-346.00)	170.50 ± 58.21 (124.00-264.00)	0.57*
TSH^a^ (mIU/mcL)	1.90 (1.30–2.99)	2.75 (1.82–3.56)	1.87 (1.20–2.99)	1.72 (0.92–2.42)	0.05^¶^
e-GFR^a^ (mL/min)	71.00 (57.00–87.00)	70.00 (56.30–87.00)	70.00 (57.00–85.00)	74.10 (59.60–93.20)	0.73^¶^

^**a**^ mean ± standard deviation (minimum-maximum) ^b^ median (Q1-Q3) * One-way ANOVA ^¶^ Kruskal-Wallis testFBS, fasting blood sugar; HbA1C, hemoglobin A1C; BUN, blood urea nitrogen; Total-C, total cholesterol; HDL-C, high-density lipoprotein cholesterol; LDL-C, low-density lipoprotein cholesterol; AST, aspartate aminotransferase; ALT, alanine aminotransferase; ALP, alkaline phosphatase; TSH: thyroid stimulating hormone; e-GFR: estimated glomerular filtration rate

body composition parameters for all participants are presented in Table 3. The mean FFM index demonstrated an ascending trend from the ABI < 0.9 subgroup towards the ABI > 1.3 subgroup, mirroring the trend observed for mean ABI itself; however, this difference in FFM across subgroups was not statistically significant. A similar non-significant trend was observed for TBW, MM, and SFA indices. Conversely, no consistent pattern was discernible for the mean AFR, VFA, and VSR indices. Overall, statistical analyses revealed no significant differences for any of the assessed body composition parameters between the ABI subgroups.

**Table 3:** Body composition parameters of the study population

BIA indices	Total (*n* = 100)	ABI, mean ± SD			*p*-Value
		< 0.9 (*n* = 11)	0.91–1.3 (*n* = 77)	> 1.3 (*n* = 12)	
FFM^a^ (kg)	48.10 (29.80–55.50)	52.10 (42.90–56.80)	48.00 (39.85-55.00)	48.15 (43.15–57.1)	0.79*
TBW^b^ (L)	35.77 ± 6.97 (21.90–53.40)	34.84 ± 5.76 (25.5–46.3)	35.64 ± 7.14 (21.9–53.4)	37.42 ± 7.13 (28.0-45.2)	0.64^¶^
MM^b^ (kg)	45.66 ± 9.24 (27.40–71.00)	44.50 ± 7.59 (32.90–59.40)	45.51 ± 9.54 (27.40–71.00)	47.66 ± 9.00 (36.00-58.10)	0.69^¶^
AFR^b^	0.92 ± 0.05 (0.81–1.04)	0.92 ± 0.04 (0.85–1.04)	0.92 ± 0.05 (0.82–1.03)	0.92 ± 0.04 (0.83–0.98)	0.98^¶^
VFA^a^ (cm^2^)	105.60 (90.40-124.25)	97.20 (90.82-116.93)	109.00 (90.20-128.40)	104.95 (83.20-114.33)	0.44*
SFA^b^ (cm^2^)	214.44 ± 99.08 (2.50–500.00)	184.57 ± 114.33 (70.10-458.90)	217.43 ± 92.28 (2.50–500.00)	223.37 ± 99.76 (43.60-401.50)	0.55^¶^
VSR^a^	0.50 (0.40–0.65)	0.66 (0.39-83.00)	0.49 (0.40–0.60)	0.48 (0.38–0.84)	0.92^¶^

^a^ median (Q1-Q3) ^b^ mean ± standard deviation (minimum-maximum) * Kruskal-Wallis tests ^¶^ One-way ANOVAFFM, Fat free mass; TBW, Total body water; MM, Muscle mass; AFR, Abdominal fat ratio; VFA, Visceral fat area; SFA, Subcutaneous fat area; VSR, visceral to subcutaneous fat ratio

To more specifically address the differences between normal patients and those with PAD, we omitted those with ABI > 1.3 from our further considerations. The analysis of demographic and clinical data for patients with normal ABI versus low ABI (defined as < 0.9 and indicative of PAD) is presented in Table 4. The two groups had no significant difference in any demographic or clinical characteristics.

**Table 4:** Demographic and clinical characteristics of patients with normal and low ABI

Characteristic	Sample (*n* = 88)	ABI subgroups		*p*-Value
		≤ 0.9 (*n* = 11)	0.91–1.3 (*n* = 77)	
Age^a^ (years)	62.67 ± 8.80	62.82 ± 11.64	62.65 ± 8.41	0.95*
Male^b^	42 (47.70)	6 (54.50)	36 (46.80)	0.63^¶^
ABI-R^c^ (mmHg)	1.05 (0.98–1.16)	0.84 (0.81–0.91)	1.08 (1.01–1.17)	< 0.001^§^
ABI-L^c^ (mmHg)	1.00 (0.94–1.09)	0.83 (0.75–0.85)	1.02 (0.98–1.11)	< 0.001^§^
Weight^a^ (kg)	73.82 ± 12.69 (47.50–111.00)	70.24 ± 9.91 (54.6-110.4)	74.33 ± 13.01 (47.5–111.0)	0.32*
Height^a^ (cm)	162.65 ± 10.31 (138.00-187.00)	161.09 ± 10.79 (142.0-178.0)	162.88 ± 10.29 (138.0-187.0)	0.59*
BMI^c^ (kg/m^2^)	27.98 ± 4.42 (18.28–46.20)	27.43 ± 4.26 (21.06–38.20)	28.09 ± 4.45 (18.28–46.20)	0.56*
Waist Circumference^c^ (cm)	90.60 (85.90–96.90)	88.7 (86.93-96)	91.1 (85.9–97)	0.32^§^
SBP^c^ (mmHg)	120.00 (110.00-135.00)	117.5 (115–135)	120 (110-131.5)	0.49 ^§^
DBP^c^ (mmHg)	80.00 (70.00–85.00)	77.5 (75-88.5)	80 (70–85)	0.96^§^
Diabetes duration^c^ (years)	12 (8-18.50)	13 (8–25)	12 (8–18)	0.26^§^
Smoking^b^	3 (3.40)	0 (0.00)	3 (3.90)	0.99^¶^
HTN^b^	42 (47.70)	7 (63.60)	35 (45.50)	0.34^¶^
Thyroid^b^	18 (20.50)	3 (27.30)	15 (19.50)	0.69^¶^
Dyslipidemia^b^	5 (5.70)	0 (0.00)	5 (6.50)	0.99^¶^
Cancer^b^	5 (5.70)	0 (0.00)	5 (6.50)	0.88^¶^
Cardiac events^b^	16 (18.20)			
Angioplasty	10 (11.40)	1 (9.10)	9 (11.70)	0.83^¶^
Angioplasty & MI	6 (6.80)	1 (9.10)	5 (6.50)	
Surgical History^b^	15 (17.00)	1 (9.10)	14 (18.20)	0.68^¶^
Family history of disease^b^	68 (77.30)			0.13^¶^
Diabetes	44 (50.00)	4 (36.40)	40 (51.90)	
HTN	7 (8.00)	0 (0.00)	7 (9.10)	
Diabetes & HTN	15 (17.00)	1 (9.10)	14 (18.20)	
Cancer	2 (2.30)	1 (9.10)	1 (1.30)	

^a^ mean ± standard deviation (minimum-maximum) ^b^ n (%) ^c^ median (Q1-Q3)* Independent sample t test ^¶^ Chi-square analysis ^§^ Mann-Whitney U test ABI-R, Ankle Brachial Index- right arm and right leg; ABI-L, Ankle Brachial Index- left arm and left leg; BMI, Body Mass Index; SBP, Systolic Blood Pressure; DBP, Diastolic Blood Pressure; HTN, Hypertension

Respective laboratory comparisons (Table 5) showed that HDL levels were significantly higher in the normal ABI group. Furthermore, although TSH levels remained within the normal reference range for both groups, they were significantly elevated in the low ABI group. While trends were observed for lower mean e-GFR and higher mean triglycerides in the low ABI group, no other laboratory parameters differed significantly between groups.

body composition parameters (Table 6) also revealed no significant differences between the two ABI categories. Results from multivariate logistic analysis, using PAD as the outcome and ABI < 0.9 as a surrogate marker, are shown in Table 7. In both adjusted models, none of the selected variables were independently associated with PAD.

**Table 5:** Biochemical features of patients with normal and low ABI

Lab Features	Sample (n=88)	ABI subgroups		p-Value
		≤ 0.9	0.91- 1.3	
		(n=11)	(n=77)	
FBS^a^ (g/dL)	126.50 (110.00-154.00)	136.50 (92.75-151.75)	126.50 (109.75-156.25)	0.79*
HbA1C^b^ (%)	6.90 (6.40-7.75)	7.10 (6.52-7.72)	6.90 (6.30-7.90)	0.96*
Hemoglobin^b^ (g/dL)	14.35 (13.00-15.42)	15.10 (12.62-15.92)	14.00 (12.92-15.07)	0.29*
BUN^b^ (mg/dL)	17.25 (14.15-21.00)	17.00 (13.95-25.75)	17.50 (14.00-21.00)	0.57*
Creatinine^b^ (mg/dL)	1.00 (0.82-1.20)	1.08 (0.90-1.24)	1.00 (0.81-1.20)	0.48*
Total-C^b^ (mg/dL)	138.50 (127.00-159.75)	130.50 (118.50-159.75)	140.00 (127.75-161.25)	0.97*
HDL-C^b^ (mg/dL)	45.00 (36.00-50.00)	36.50 (31.25-41.50)	46.00 (38.00-52.00)	0.02*
LDL-C^a^ (mg/dL)	75.94 ± 21.23 (28.00-127.00)	75.26 ± 22.16 (37.00-122.00)	75.89 ± 20.44 (28.00-127.00)	0.95^¶^
Triglycerides^b^ (mg/dL)	124.00 (98.00-155.50)	134.50 (107.50-312.25)	123.00 (97.00-153.00)	0.79*
25-OH Vit D^b^(ng/mL)	35.60 (27.92-45.00)	29.40 (26.00-33.75)	37.85 (28.00-48.00)	0.43*
AST^b^ (mg/dL)	20.00 (17.00-24.50)	21.50 (14.50-28.80)	20.00 (17.00-24.00)	0.14*
ALT^b^ (mg/dL)	21.00 (15.00-30.00)	28.50 (20.25-37.00)	20.00 (15.00-27.00)	0.35*
ALP^a^ (mg/dL)	169.63 ± 67.60 (54.00-346.00)	191.56 ± 57.64 (56.00-273.00)	165.55 ± 68.71 (54.00-346.00)	0.34^¶^
TSH^b^ (mIU/mcL)	2.00 (1.37-2.99)	2.75 (1.82-3.56)	1.87 (1.20-2.99)	0.014*
e-GFR^b^ (mL/min)	71.00 (57.00-86.00)	70.00 (56.30-87.00)	70.00 (57.00-85.00)	0.49*

^a^mean ± standard deviation (minimum-maximum)^b^median (Q1-Q3)* Mann-Whitney U test^¶^Independent sample t test FBS, fasting blood sugar; HbA1C, hemoglobin A1C; BUN, blood urea nitrogen; Total-C, total cholesterol; HDL-C, high-density lipoprotein cholesterol; LDL-C, low-density lipoprotein cholesterol; AST, aspartate aminotransferase; ALT, alanine aminotransferase; ALP, alkaline phosphatase; TSH: thyroid stimulating hormone; e-GFR: estimated glomerular filtration rate

**Table 6:** Body composition parameters of patients with normal and low ABI

Body composition parameters	Sample (n=88)	ABI subgroups		p-Value
		< 0.9(n = 11)	0.91- 1.3(n = 77)	
FFM^a^ (kg)	48.10 (40.60-55.50)	52.10 (42.90-56.80)	48.00 (39.85-55.00)	0.61*
TBW^b^ (L)	35.54 ± 6.95 (21.90-533.40)	34.84 ± 5.76 (25.5-46.3)	35.64 ± 7.14 (21.9-53.4)	0.72^¶^
MM^b^ (kg)	45.38 ± 9.29 (27.40-71.00)	44.50 ± 7.59 (32.90-59.40)	45.51 ± 9.54 (27.40-71.00)	0.74^¶^
AFR^b^	0.92 ± 0.05 (0.82-1.04)	0.92 ± 0.04 (0.85-1.04)	0.92 ± 0.05 (0.82-1.03)	0.86*
VFA^a^ (cm^2^)	104.80 (90.00-125.80)	97.20 (90.82-116.93)	109.00 (90.20-128.40)	0.19*
SFA^b^ (cm^2^)	213.18 ± 99.51 (2.50-500.00)	184.57 ± 114.33 (70.10-458.90)	217.43 ± 92.28 (2.50-500.00)	0.33^¶^
VSR^a^	0.50 (0.40-0.65)	0.66 (0.39-83.00)	0.49 (0.40-0.60)	0.92*

^a^ median (Q1-Q3) ^b^ mean ± standard deviation (minimum-maximum) * Mann-Whitney U test ^¶^ Independent sample t test FFM, Fat free mass; TBW, Total body water; MM, Muscle Mass; AFR, Abdominal fat ratio; VFA, Visceral fat area; SFA, Subcutaneous fat area; VSR, Visceral to subcutaneous fat ratio

## Discussion

To our knowledge, this study represents one of the first few investigations to examine the relationship between a panel of body composition parameters and the ankle-brachial index (ABI), while concurrently evaluating their associations with a range of clinical and paraclinical factors in a diabetic population. Analysis of data from 100 patients revealed no significant association between the investigated body composition parameters and ABI (p-Value > 0.05). Further statistical analyses, categorizing patients into two and three subgroups based on standard ABI cut-offs (first comparison: ABI < 0.9, 0.91–1.3, > 1.3; second comparison: ABI 0.9–1.3 vs. ABI < 0.9), also showed no significant results (Tables 3 and 6). However, the analyses revealed a significant difference in the frequency of HTN across ABI categories (Table 1). Contrary to expectations, in the subset of 88 patients with normal or reduced ABI, no significant difference in HTN frequency was observed (p-Value > 0.05). This discrepancy may be attributable to the study’s relatively small sample size and the fact that only approximately 11% of all participants had ABI values compatible with a diagnosis of PAD. The study found no significant differences across ABI categories in terms of other clinical and background variables, including age, sex, weight, height, BMI, waist circumference, systolic and diastolic blood pressure levels, diabetes duration, smoking status, or history of thyroid disease, dyslipidemia, malignancy, cardiovascular events, recent surgery, or familial metabolic diseases. Regarding the multivariable models, in model A, neither age, hypertension, nor the muscle mass (MM) was independently associated with PAD. Likewise, in Model B, no independent associations were observed between PAD and age, hypertension, and the visceral fat area (VFA) after adjustment for age and hypertension (Table 7).

**Table 7:** Multivariable binary logistic regression analyses of representative body composition parameters associated with peripheral arterial disease (PAD; ABI < 0.90)

Models	OR (95%CI)	p-Value
**Model A**		
Age	1.001 (0.92-1.08)	0.98
Hypertension	1.60 (0.44-5.78)	0.46
MM	1.02 (0.96-1.09)	0.43
**Model B**		
Age	0.99 (0.92-1.08)	0.98
Hypertension	1.56 (0.42-5.71)	0.50
VFA	1.00 (0.98-1.01)	0.97

ABI: Ankle-Brachial Index; OR, odds ratio; 95%CI: 95% confidence interval; MM, Muscle mass; VFA, Visceral fat area

A recent Ecuadorian study reported that BIA-derived lean tissue parameters, including skeletal muscle mass, intracellular water, and phase angle, were independently associated with a lower risk of diabetic vascular complications, whereas visceral fat area was associated with increased risk [43]. In contrast, our study did not identify independent associations between representative BIA parameters and PAD. This discrepancy may be explained by differences in the vascular outcomes evaluated, statistical methodology (including the use of principal component analysis to derive composite body composition patterns), and the limited number of PAD events in our study.

Kim et al. investigated the relationship between body composition and insulin resistance using dual-energy X-ray absorptiometry (DXA). They found that increased fat mass, regardless of muscle mass level, was associated with a higher risk of metabolic abnormalities, suggesting that excess adiposity plays a greater role than muscle mass in metabolic health. However, their findings are not directly comparable with the present study because they evaluated metabolic outcomes using DXA rather than PAD using BIA [48]. The discrepancy between our findings and those of Kim et al. may be explained by several factors, including differences in study outcomes (PAD versus metabolic abnormalities), body composition assessment methods (BIA versus DXA), study populations, and statistical power. In particular, the limited number of PAD events in our study may have reduced the ability to detect independent associations between body composition parameters and PAD.

Similarly, a large cross-sectional analysis of the NHANES cohort investigated the association between body composition and PAD using ABI as the outcome measure [42]. The authors found that greater total and trunk fat mass were independently associated with PAD, whereas no significant associations were observed for fat-free mass or body fluid volume. In contrast, our study did not identify independent associations between representative BIA-derived body composition parameters and PAD. The differing findings may be attributable to differences in study populations, body composition assessment methods (DXA versus BIA), and the substantially smaller number of PAD events in our cohort, which may have limited the statistical power to detect independent associations.

Furthermore, a 2017 systematic review and meta-analysis by Hajibandeh et al. of 43 observational cohort studies (*n* = 94254) found that a low ABI (< 0.9) was a strong independent predictor of all-cause mortality, cardiovascular mortality, stroke, and myocardial infarction [49]. The increased risk pattern held true even in asymptomatic individuals and those with existing comorbidities like diabetes. This highlights the ABI’s role as a marker of atherosclerosis. The lack of significant association between ABI and other clinical metrics in our study may be due to limited sample size and potential referral bias, as participants were likely under closer specialist supervision, potentially leading to better diabetes control.

Regarding laboratory parameters, patients with PAD (ABI < 0.90) had significantly lower HDL-C levels and higher TSH levels than those without PAD, whereas no significant differences were observed for other biochemical markers. The association between low HDL-C and PAD is consistent with previous studies identifying reduced HDL-C as an independent marker of increased cardiovascular and cardiometabolic risk [50–54]. Likewise, the higher TSH levels observed in our PAD group are in agreement with reports describing an increased prevalence of subclinical hypothyroidism among patients with PAD [55], although conflicting findings have also been reported [56]. Emerging evidence further suggests that thyroid dysfunction may contribute to PAD through adverse lipid metabolism and chronic low-grade inflammation, while cardiovascular benefits have been observed following levothyroxine therapy in selected patients with subclinical hypothyroidism [57–63].

This study had some limitations that should be considered when interpreting the findings. Besides the basic limitations of a cross-sectional study, the sample size was modest, especially in subgroups such as patients who met the ABI criteria for peripheral arterial disease, which may have reduced the statistical power. Recruitment from a single tertiary center introduces the possibility of referral bias and may have selected patients with more complex conditions or greater access to care. Lastly, results could have been more accurate by including inflammatory markers such as C-reactive protein. Future research may address these limitations through well-designed prospective studies with larger and more diverse populations. Multicenter investigations based on community cohorts would improve generalizability and allow longitudinal assessment of the relationship between body composition and vascular status. Further work is needed to clarify the potential roles of paraclinical markers in diabetic peripheral arterial disease.

## Conclusion

BIA-derived body composition parameters were not independently associated with peripheral arterial disease in this cohort of high-risk patients with type 2 diabetes mellitus. However, lower HDL-C and higher TSH levels were observed among patients with PAD, suggesting potential metabolic and endocrine correlates of vascular disease. Further large-scale prospective studies are warranted to clarify the role of body composition and biomarkers in PAD risk assessment.

## Data Availability

The data that support the findings of this study are available from Imam Reza Clinical Research Unit, Mashhad University of Medical Sciences, but restrictions apply to the availability of these data, which were used under license for the current study, and so are not publicly available. Data are however available from the authors upon reasonable request and with permission of Imam Reza Clinical Research Unit.

## Abbreviations

T2DM: Type 2 diabetes mellitus
PAD: Peripheral arterial disease
ABI: Ankle-brachial index
BIA: Bioelectric impedance analysis
DXA: Dual X-ray absorptiometry
CT: Computed tomography
MRI: Magnetic resonance imaging
ABI-R: Ankle-brachial index (Right)
ABI-L: Ankle-brachial index (Left)
FFM: Fat-free mass
MM: Muscle mass
TBW: Total body water
WC: Waist circumference
AFR: Abdominal fat ratio
VFA: Visceral fat area
SFA: Subcutaneous fat area
VSR: Visceral to subcutaneous fat ratio
FBS: Fasting blood sugar
HbA1c: Glycated hemoglobin
SBP: Systolic blood pressure
DBP: Diastolic blood pressure
HDL: High-density lipoprotein cholesterol
LDL: Low-density lipoprotein cholesterol
TG: Triglycerides
BUN: Blood urea nitrogen
e-GFR: estimated glomerular filtration rate
AST: Aspartate aminotransferase
ALT: Alanine aminotransferase
ALP: Alkaline phosphatase
WBC: White blood cell count
Hb: Hemoglobin
Plt: Platelet count
TSH: Thyroid-stimulating hormone
